# Post–Coronavirus Disease 2019 Aging Agenda for 2021 and Beyond

**DOI:** 10.1093/ppar/praa025

**Published:** 2020-09-04

**Authors:** Gretchen E Alkema

**Affiliations:** The SCAN Foundation, Long Beach, California

**Keywords:** Complex care, Retirement security, Health security, COVID-19

On January 26, 2020, the Centers for Disease Control and Prevention confirmed California’s first case of coronavirus disease 2019 (COVID-19) in a mid-50s male who had traveled to Wuhan, China, the outbreak’s global epicenter. As the third confirmed case in the United States, he was released from the hospital in good condition on February 1, 2020, and was relegated to home isolation (Wheeler, 2020). In the months that followed, the United States identified nearly 5.1 million cases, resulting in nearly 164,000 deaths as of August 12, 2020. (Centers for Disease Control and Prevention, 2020).

COVID-19’s infiltration into every American, as well as global, community has upended daily life across our nation’s social, demographic, political, economic, and certainly health-care landscapes. COVID-19’s infection and death rates continue to rise, exposing the underlying health-care and economic security vulnerabilities for all Americans, and most particularly older adults, those living with complex care needs, and their family caregivers.

Here is what is known about COVID-19’s health-related impact in its first 6 months in America (Centers for Disease Control and Prevention, 2020; Centers for Medicare & Medicaid Services, 2020; Collins, 2020; Ford et al., 2020; Stokes et al., 2020):

- Nearly 80% of all COVID-19–related deaths are among those age 65 and older, with nearly 40% in nursing facilities.
- Hospitalizations are six times higher and deaths 12 times higher for those with reported underlying severe conditions, such as cardiovascular disease and diabetes.
- Communities experiencing economic and social inequities along with health disparities have been more severely impacted. For example, Black Americans represent 13% of the population, yet comprise 23% of COVID-19–related deaths. In every age decile, they have died from COVID-19 at roughly the same rate as Whites who are more than a decade older.

Public- and private-sector leaders have been shaken by the virus’ ability to spread quickly and quietly. Anticipating the worst with comparisons to the 1918 influenza pandemic, the response to COVID-19 fundamentally altered national and global economies in ways not seen outside of a world war. Given that the only specific population-scale prevention interventions are heightened hygiene practices, consistent facial covering, and physical distancing, these actions have nearly halted all aspects of modern life in ways that would have been difficult to fathom during a 2019 Thanksgiving dinner conversation. An integrative gerontological perspective reminds us that the biological, psychological, and social/economic factors of the COVID-19 crisis will have lasting impacts at both the individual and population levels (Alkema & Alley, 2020).

A crisis of this magnitude is a blending of danger and opportunity. The danger is apparent in measurable items, such as infection rates, mortality statistics, social unrest, and economic output, as well as less measurable ways, such as human isolation, social and economic disengagement, and fear of the future. However, significant opportunities to drive change in all aspects of the aging trajectory to radically improve quality of life and meet person- and family-centered needs are also meaningfully present. Take health-care spending, for example. It declined 24% in April compared to the previous year, largely due to the cancellation of elective procedures and low patient volumes (Altarum, 2020). This signals severe danger for a key sector that has been a mainstay of the U.S. economy even during recovery from the 2008 financial meltdown. However, from a person- and family-centered perspective, care delivery system leaders and payers are now seeing the raw power of consumer engagement and disengagement in health care, even when forced. Forward-thinking leaders who abandon the notion of health care as a commodity, and instead embrace it as a process for individual and societal wellness and equity, will transform care systems in ways that exceed quality targets and enhance organizational sustainability.

So what do we do now? What actions can the highest levels of leadership—starting with the President in January 2021—take to catalyze the opportunity of this prescient moment in order to create a world where all people—regardless of age, life circumstance, or ability—live well in the place they call home across their life course?

What actions can the highest levels of leadership—starting with the President in January 2021—take to catalyze the opportunity of this prescient moment in order to create a world where all people—regardless of age, life circumstance, or ability—live well in the place they call home across their life course?

Fundamental roles of government, as headed by the chief executive, are often described as protector, provider, and talent investor to meet the needs for all individuals, families, and society (Slaughter, 2017). Here are five ways the 2021–2024 president should lead in this transformational moment.

1. Federal leadership: appoint a White House–level leader on aging. Name and give authority to a national leader who will build solutions for aging Americans across all domestic policy areas. Aging is everybody’s business, affecting nearly all cabinet-level departments, agencies, and affiliates. Visible federal leadership is vital to prioritize issues affecting older Americans and family caregivers of today and tomorrow, and take steps to strengthen our social and economic infrastructure so that it works well for all Americans regardless of age, background, or culture. This person should be a White House–level leader charged with problem-solving and cross-cutting coordination between the Domestic Policy Council, National Economic Council, Chief of Staff, Departmental Secretaries and related Administrators, Congressional and community leaders, and others.
2. State leadership: call on each governor to develop a Master Plan for Aging. Every state has acutely felt the impact of COVID-19 as it relates to an aging population. Forward-thinking leaders have acted by reworking regulations to minimize the death rate among care facility residents, revamping home- and community-based services programs to keep more at-risk individuals away from high-density environments where the virus breeds, and incorporating state unit on aging leadership into processes for emergency planning and the public health infrastructure. The COVID-19 crisis shows the importance of taking lessons from this moment and decisively planning for the needs of older Americans of today and tomorrow through a thoughtful, comprehensive, and outcomes-oriented strategy, called a Master Plan for Aging (Chernof, 2019). California and other states are developing or have a Master Plan for Aging, which serves as a crosscutting blueprint by state government—in collaboration with local communities, private organizations, and philanthropy—to build environments that promote age-friendly living and engagement (Together We Engage, 2020). Several states have used a Master Plan for Aging to set a long-range vision and direction to recalibrate care delivery, state oversight, and fiscal prioritization toward home- and community-based care (The SCAN Foundation, 2020). It is time that each state follows suit to ensure that an aging lens is prioritized across all departments in the administration and the legislature.
3. Local infrastructure: bridge the divide between individual/family basic needs and essential services. COVID-19 has reminded humanity about the fundamentals of daily life for the 21^st^ century, which include food, shelter, and Internet access. However, millions seek to survive without these necessities each day, and the pandemic illuminated longstanding infrastructure inequalities in stark detail. Leadership must demand the removal of key infrastructure “deserts” that exist in impoverished, under-resourced, and often rural communities. The solution is to pave the way for affordable, meaningful, and life-supporting access to food, housing, or high-speed Internet in every populated zip code, to nurture work, play, and family life.
4. Health security: amplify value-based care delivery and payment. It is time to abandon the consumption model of health care and recalibrate it to be laser focused on outcomes that maximize individual and societal wellness and equity. Below are four specific examples that are ripe for this level of transformation. First, leverage the wide range of federal and state regulatory flexibilities in Medicare and Medicaid that were offered during the early months of COVID-19 to maximize person-centered care toward high-quality outcomes (Alkema, 2004; Anthony et al., 2020; Podulka & Blum, 2020; The SCAN Foundation, 2020). Second, ramp up virtual care (e.g., telehealth) structures, processes, payment, accountability, workforce scope of practice, and quality control to serve adults of all ages with complex care needs in the most convenient location and time for them. Third, revise Medicare Advantage payment systems and regulation to radically expand supplemental benefit offerings that enable a whole range of home-based care, including deeper use of remote monitoring and other digital technologies to maximize health outcomes (ATI Advisory & LTQA, 2020). Fourth, accelerate federal and state efforts to align and integrate Medicare and Medicaid, as these programs serve some of the most vulnerable members of our aging society; this acceleration would largely include deeper federal incentives for states and health plans to participate (Soper et al., 2020).
5. Economic security: recalibrate labor force engagement for family caregivers and retirement security for all workers. COVID-19 has fundamentally changed the way in which people work and prepare for retirement, while also rocking the economic stability and future retirement trajectory of late Boomers, GenXers, and early Millennials who have suffered job shrinkage and loss. The United States needs to bolster and stabilize Social Security for current and future generations, while creating pathways to cover future short- and long-term care costs as people age. Now is the time to endorse employment security for working family caregivers, so they can care for loved ones with complex care needs without eroding their own future economic security. One example is to recognize and amplify innovative workplace/human-resource policies in public- and private-sector employment that maximize family caregiving engagement while maintaining workplace productivity.

Now is the time where leadership, grounded by clear and expansive vision and commensurate strategy, can convert this terrible global pandemic moment into the foothold of a new world. The only limitations are the bounds of our creativity and willingness to meet this moment head on.

## Funding

None delared.

## Conflict of Interest

None delared.
